# Domain-specific cognitive screening in acute first-ever stroke: a comparative study of the Oxford Cognitive Screen (OCS) and ACE-III

**DOI:** 10.3389/fnhum.2025.1678230

**Published:** 2026-01-12

**Authors:** Onur Tanglay, Dhruv Jhunjhnuwala, William Huynh

**Affiliations:** 1 Faculty of Medicine and Health, South West Sydney Clinical School, UNSW Sydney, Liverpool, NSW, Australia; 2 Faculty of Medicine and Health, School of Medical Sciences, The University of Sydney, Sydney, NSW, Australia; 3 Faculty of Medicine and Health, Randwick Clinical School, UNSW Sydney, Randwick, NSW, Australia

**Keywords:** ACE-III, cognition, cognitive screening, OCS, post-stroke cognitive impairment, stroke

## Abstract

**Introduction:**

Post-stroke cognitive impairment (PSCI) is common and often under-recognized, particularly in the acute phase. Most cognitive screening tools provide only a global score, overlooking domain-specific deficits that influence functional recovery. The Addenbrooke’s Cognitive Examination-III (ACE-III) is a comprehensive cognitive test whose utility for acute stroke patients remains under-studied. This study evaluated the diagnostic performance of the ACE-III against the stroke-specific Oxford Cognitive Screen (OCS) in detecting PSCI following first-ever stroke in the acute period.

**Methods:**

Patients with first-ever stroke and no history of cognitive impairment were prospectively assessed within seven days of onset using both the OCS and ACE-III. PSCI was defined by impairment in one or more cognitive domains on the OCS. The discriminatory capacity of the ACE-III for detecting PSCI was examined, and associations between specific cognitive deficits and functional dependence were analyzed.

**Results:**

The OCS detected PSCI in 70% of the 30 patients that were recruited. The ACE-III demonstrated good discriminatory capacity (AUC = 0.897); however, it failed to detect PSCI in five patients identified by the OCS, and misclassified two aphasic but cognitively intact patients as impaired. Two patients classified as impaired on ACE-III were deemed cognitively intact by OCS, underscoring its limitations in stroke populations. Standard and stroke-specific ACE-III cut-offs demonstrated suboptimal accuracy for acute screening.

**Conclusion:**

While ACE-III performs well at the group level, it may miss relevant cognitive impairment in the acute stroke setting. Domain-based, stroke-specific tools such as the OCS more reliably detect deficits and may offer greater clinical utility for early cognitive profiling and rehabilitation.

## 1 Introduction

Despite improvements in hyperacute therapy and focus on reducing door-to-needle time, post-stroke cognitive impairment (PSCI) remains a frequent and disabling consequence of acute stroke (Gallucci et al., 2024). Patients with PSCI suffer poorer functional outcomes, impaired quality of life, and a higher risk for recurrent stroke (Levine et al., 2015; Obaid et al., 2020). PSCI is classified as a form of vascular cognitive impairment (VCI), which in turn encompasses a broad spectrum of cognitive dysfunction associated with any amount of cerebrovascular pathology (Melkas et al., 2014; van der Flier et al., 2018). Patients with PSCI typically exhibit dysfunction in a single cognitive domain without direct loss of independence in activity of daily living, with multi-domain impairment occurring less frequently (Cumming et al., 2013; Lo et al., 2019; Melkas et al., 2014; Srikanth et al., 2003; van der Flier et al., 2018).

Incidence of PSCI has been reported to range from 39% to more than 70% (Leśniak et al., 2008; Lo et al., 2019; Ma et al., 2025; Pendlebury and Rothwell, 2009). This is highest in the acute period, and notably, these elevated rates are present even among patients with mild neurological deficits (Gallucci et al., 2024; Milosevich et al., 2024). The substantial variation in incidence can be attributed to the heterogeneity of study cohorts, encompassing inclusion of recurrent-strokes and premorbid cognitive impairment, individual patient factors, assessment timing, and assessment tool (Sexton et al., 2019; Sun et al., 2014).

Despite the clinical significance and high prevalence of PSCI, there remains no universally accepted gold standard for cognitive screening in acute stroke populations. The Mini-Mental State Examination (MMSE) and Montreal Cognitive Assessment (MoCA) continue to serve as the most widely employed bedside instruments, yet both demonstrate substantial limitations when applied to stroke patients (Kosgallana et al., 2019; Nijsse et al., 2017). Importantly, they do not assess domain-specific cognitive impairment which is necessary for targeted rehabilitation (Demeyere et al., 2016). Furthermore, they were not designed to evaluate common stroke-specific cognitive deficits such as aphasia, visual loss, neglect, or apraxia; all of which contribute significantly to stroke-related disability (Cumming et al., 2013; Lees et al., 2017; Melkas et al., 2014; Nasreddine et al., 2005).

The limitations of general cognitive screens have catalyzed the development of the stroke-specific Oxford Cognitive Screen (OCS). It is structured to minimize the confounding effects of upper limb weakness, aphasia, and neglect whilst providing a “visual snapshot” of the individual cognitive domains most commonly affected by stroke, namely attention and executive function, language, memory, number processing, and praxis (Demeyere et al., 2015). Validated across multiple languages, the OCS consistently demonstrates superior sensitivity compared to the MMSE and MoCA (Demeyere et al., 2015; Demeyere et al., 2016). Recent evidence also supports the prognostic value of the OCS, with acute screening predicting functional outcomes at 6 months post-stroke (Milosevich et al., 2024; Milosevich et al., 2025).

The Addenbrooke’s Cognitive Examination-III (ACE-III) represents an alternative comprehensive cognitive screening approach, originally developed as an extension of the MMSE to detect global non-vascular cognitive impairment, with the addition of domain-specific sub-scores (Mioshi et al., 2006). There has been mixed findings on the utility of the ACE-III. Some studies have suggested, especially with a lower stroke-specific cut-off (< 82/100), the ACE-III may be on par with the MoCA (Ma et al., 2025), while others have found its accuracy to be suboptimal (Lees et al., 2017). Crucially, no study has yet validated the ACE-III against a dedicated, stroke-specific tool like the OCS.

The comparative effectiveness of comprehensive general screens vs. stroke-specific instruments remains insufficiently characterized, particularly in the acute post-stroke period when cognitive assessment can meaningfully inform discharge planning and rehabilitation strategies. Furthermore, contemporary stroke populations increasingly present with milder neurological deficits due to advances in acute management, yet cognitive impairment rates remain persistently high, highlighting the disconnect between traditional stroke severity measures and cognitive outcomes. This evolving clinical landscape necessitates rigorous evaluation of cognitive screening approaches to ensure optimal detection of impairments that impact functional recovery and long-term outcomes.

The current investigation addresses these knowledge gaps by providing the first direct comparison of the ACE-III and OCS in an exclusively acute, first-ever stroke cohort free from prior cerebrovascular or cognitive disorders. Through prospective recruitment within seven days of stroke onset, this study evaluates the diagnostic performance of both instruments for detecting overall and domain-specific PSCI, examines the prognostic significance of identified impairments for functional independence, and provides updated prevalence estimates in the context of contemporary acute stroke care.

## 2 Materials and methods

### 2.1 Study design and participants

A prospective observational cohort study of patients admitted to the acute stroke units at two centers, the Prince of Wales and St George Hospitals in Sydney, New South Wales was conducted. Patients were recruited consecutively to assess for PSCI within seven days of a first-ever MRI-confirmed ischemic stroke or intraparenchymal hemorrhage. Patients were excluded if they had a background of previous stroke or if neuroimaging detected the presence of prior cerebrovascular infarcts. Patients with a background of confirmed or suspected cognitive impairment were also excluded. Written informed consent was obtained from each patient. Ethical approval by the human research ethics committee of the South-Eastern Sydney Local Health District was obtained (2020/ETH01489). The STARD checklist was used in preparation of the manuscript (Bossuyt et al., 2015).

### 2.2 Clinical variables

Baseline characteristics recorded included the patient’s age, sex, handedness, and level of education. According to the neuroimaging results, stroke type was classified into either ischemic or hemorrhagic, while stroke lesion lateralization was recorded as either left-sided, right-sided, bilateral, or cerebellar. Ischemic strokes were further categorized utilizing the Bamford Classification into either total anterior circulation infarcts (TACI), partial anterior circulation infarcts (PACI), lacunar infarcts (LACI), or posterior circulation infarcts (POCI) (Bamford et al., 1991). The TOAST classification was used to categorize the underlying etiology of ischemic strokes into either large artery atherosclerosis, small vessel occlusion, cardio-embolism, stroke of other determined etiology, or stroke of undetermined etiology (Adams et al., 1993).

The NIHSS was used to score the clinical severity of stroke on admission, with total scores categorized as normal (NIHSS 0), mild stroke (NIHSS 1–4), moderate stroke (NIHSS 5–15), moderate-severe stroke (NIHSS 16–20), and severe stroke (NIHSS 21–42) (Lyden et al., 1994). The use of acute stroke interventions, including conservative management, thrombolysis, and endovascular clot retrieval, were recorded. The Barthel Index (BI) was used to measure the level of functional independence with activities of daily living on day seven of admission, with scores further categorized as either independent (95–100), or dependent (0–94) (Mahoney and Barthel, 1965).

### 2.3 Neuropsychological assessment

The OCS contains tasks that are grouped into stroke-specific cognitive domains (Table 1). Domain impairment was recorded if a patient scored below the included cut-off score on any one of its associated tasks. PSCI was defined in patients that exhibited impairment in ≥ one cognitive domain, and patients were further categorized into severe PSCI if they exhibited impairments in ≥ three domains. To maximize inclusivity, patients who could not be tested for specific tasks due to functional impairments were categorized as impaired and included in all analyses.

**TABLE 1:** OCS and ACE-III tasks mapped to stroke-specific cognitive domains.

Stroke-specific domains	OCS tasks	ACE-III tasks (maximum score)
Attention	Cancellation Object neglect Spatial neglect Executive function trails	Verbal fluency (14) Visuospatial abilities (16)
Language	Picture naming Semantics Sentence reading	Sentence writing (2) Word repetition (2) Sentence repetition (2) Picture naming (12) Picture pointing (4) Reading (1)
Memory	Orientation Verbal recall and recognition Episodic recognition	Orientation (10) Three-item recognition (3) Three-item recall (3) Address recognition (7) Retrograde memory (4) Address recall (12)
Number processing	Number writing Calculation	Serial subtraction (5)
Praxis	Gesture imitation	Motor comprehension (3)

OCS, Oxford Cognitive Screen; ACE-III, Addenbrooke’s Cognitive Examination III. Adapted from Quinn et al. (2018).

Unlike the OCS, the ACE-III provides a total score for global cognitive function. Patients that scored < 88/100 were categorized as having PSCI. A proposed stroke-specific cut-off score of < 82/100 for PSCI was also recorded for comparison against the standard cut-off (Kessels et al., 2017; Patel et al., 2002). While the ACE-III’s tasks are also arranged into domain sub-scores, they do not directly correspond to those being assessed by the OCS, and no normative data or established cut-offs are currently available. To enable domain-level comparison, individual ACE-III tasks were re-mapped to the five OCS cognitive domains (Table 1). This process was guided by the framework described by Quinn et al. (2018), who classified ACE-R items (an earlier version of the ACE) into the Diagnostic and Statistical Manual of Mental Disorders (DSM) cognitive domains. Since the OCS incorporates executive function within its attention domain, tasks labeled by Quinn et al. (2018) as executive (verbal fluency) were assigned to Attention. The visuospatial tasks in the ACE-III, although categorized by Quinn et al. (2018) under perceptual-motor function, were re-mapped to the OCS attention category due to their conceptual similarity to OCS subtests assessing object and spatial neglect. Serial subtraction, classified by Quinn et al. (2018) as complex attention, was placed under Number Processing as the closest conceptual analog. Finally, motor command comprehension tasks were mapped to the OCS Praxis domain based on similarity to the praxis elements within the OCS. This re-mapping protocol has not been formally validated, however, it was derived through consensus within the research team and is presented as an exploratory method to facilitate cross-tool comparison.

The OCS and ACE-III were administered on the same day. The order of tests administered was switched for each consecutive patient, with at least 60 minutes of rest between each test to minimize fatigue. Both tests were administered by a single assessor who was blinded to all clinical information. Although complete blinding between tests did not occur, no feedback or scoring was provided during testing. The assessor therefore had limited knowledge of the patient’s performance during administration.

### 2.4 Statistical analysis

All statistical analyses were performed using IBM SPSS version 26.0 (IBM, Armonk, NY). The data were de-identified prior to analysis, and the author performing the analysis was independent from the test administration process. Receiver operating characteristic (ROC) curves and areas under the curves (AUC) were estimated to evaluate the total ACE-III score and stroke-specific ACE-III domain sub-scores’ discriminatory capacity for detecting PSCI based on a diagnosis made by the OCS. Youden’s index was utilized for each ACE-III domain sub-score to determine the optimal cut-off point that maximized sensitivity and specificity. Cut-off values derived from ROC curves were reported as continuous thresholds as the optimal Youden’s index values occur between two adjacent integer scores. These midpoints represent decision boundaries rather than actual raw scores. Thus, a threshold of < 24.5, for example, corresponds to classifying scores ≤ 24 as impaired. Confidence intervals (95% CI) were calculated for each outcome. Chi-squared and Fisher’s exact tests assessed the association between domain-specific PSCI and functional status. The *p*-values of these comparisons were adjusted using the Benjamini-Hochberg method at a false discovery rate of 0.05.

A sensitivity analysis was performed to assess whether exclusion of participants who were unable to complete tasks across all OCS domains altered the results. For each ACE-III cut-off and each remapped domain sub-score, diagnostic metrics were recalculated. Differences in sensitivity and specificity between the primary and sensitivity analyses were quantified by calculating 95% CI for the difference between two independent proportions. Effect sizes were estimated using Cohen’s *h*. For domain level analyses, ROC curves were re-estimated. There were otherwise no missing data in the OCS and ACE-III results.

Given the recruitment limitations imposed by the COVID-19 pandemic, a *post-hoc* power analysis was performed to contextualize the precision and stability of the diagnostic accuracy estimates. A conservative reference value for PSCI prevalence of 40% was used to assess the power of the observed proportion of impaired patients using a binomial estimate. The variance of the observed AUC-ROC was estimated using the Hanley-McNeil method to quantify how precisely the observed AUC differed from the null hypothesis of no discrimination (i.e., AUC 0.50). The power of the observed ACE-III sensitivity and specificity were also approximated based on a one-sample binomial distribution at two different thresholds of 60 and 80%.

## 3 Results

### 3.1 Patient characteristics

A total of 30 consecutive patients with a first-ever stroke were recruited from January to June 2021 (Table 2). No patients were excluded due to cognitive impairment, and none declined to participate in the current study. With a mean age of 67.0 ± 16.3 years, 17 (56.7%) patients were male and 13 (43.3%) were female. The vast majority (96.7%) were right-handed.

**TABLE 2:** Baseline patient characteristics.

Characteristic	Sub-characteristic	All patients (*n* = 30) (%)
Age	Mean ± SD	67.0 ± 16.3
Sex	Male	17 (56.7)
	Female	13 (43.3)
Days from stroke	Mean ± SD	3.3 ± 2.7
Handedness	Right	29 (96.7)
	Left	1 (3.3)
Level of education	Up to secondary (≤12 years)	18 (60)
	Tertiary (>12 years)	12 (40)
Stroke type	Ischemic	28 (93.3)
	Hemorrhagic	2 (6.7)
Stroke location	Left	12 (40.0)
	Right	15 (50.0)
	Bilateral	2 (6.7)
	Cerebellar	1 (3.3)
Bamford (*n* = 28)	TACI	0 (0)
	PACI	16 (57.1)
	LACI	3 (10.7)
	POCI	9 (32.1)
TOAST (*n* = 28)	Large artery atherosclerosis	7 (25.0)
	Small vessel occlusion	3 (10.7)
	Cardio-embolism	13 (46.4)
	Other etiology	0 (0)
	Undetermined etiology	5 (16.7)
NIHSS	Normal (0)	9 (30.0)
	Minor (1–4)	9 (30.0)
	Moderate (5–15)	10 (33.3)
	Moderate-severe (16–20)	1 (3.3)
	Severe (21–42)	1 (3.3)
Intervention	Conservative ± tPA	20 (66.7)
	ECR ± tPA	10 (33.3)
Barthel index	Independent (≥95)	19 (63.3)
	Dependent (<95)	11 (36.7)

A total of 28/30 (93.3%) patients had an ischemic stroke which were further categorized according to the Bamford classification into PACIs (57.1%), POCIs (32.1%), and LACIs (10.7%). There were no patients with TACIs in our sample. Utilizing the TOAST classification, the most common etiologies of ischemic strokes were cardio-embolism (46.4%), followed by large artery atherosclerosis (25.0%), undetermined etiology (16.7%), and small vessel occlusion (10.7%).

The cohort’s mean NIHSS was 5.1 ± 6.4 on admission to the stroke unit, with 18/30 (60%) patients ranging from normal to minor strokes (NIHSS ≤ 4) and 12/30 (40%) from moderate to severe strokes (NIHSS > 4). A total of 17/30 (56.7%) patients received conservative management, 3/30 (10.0%) received tPA alone, and 10/30 (33.3%) underwent ECR ± tPA. The mean overall BI score was 84.2 ± 26.3 post-intervention, with 19/30 (63.3%) patients categorized as functionally independent while 11/30 (36.7%) patients were functionally dependent.

### 3.2 Addenbrooke’s Cognitive Examination and post-stroke cognitive impairment

**FIGURE 1:**
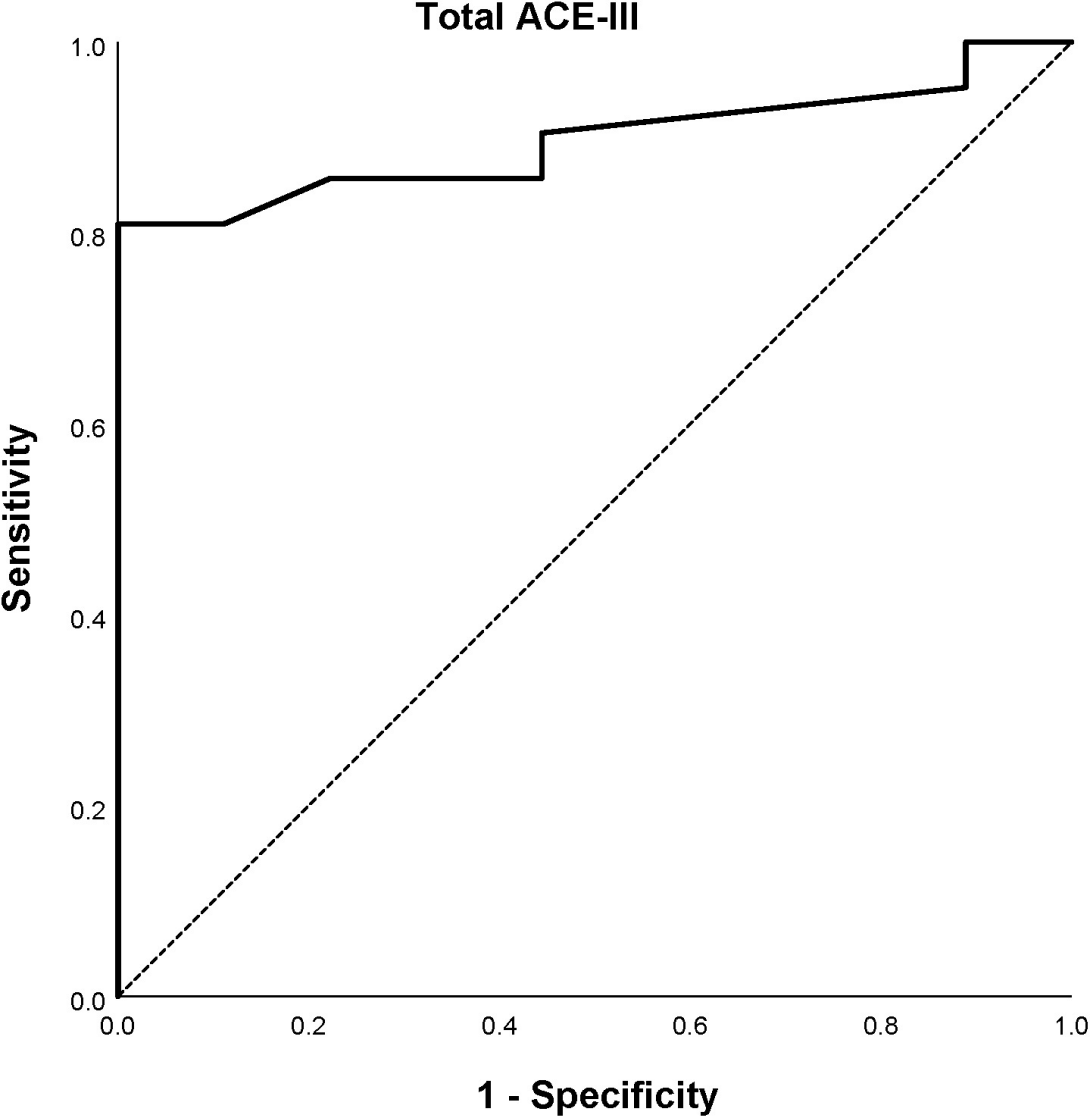
Receiver operating curve estimate for the total Addenbrooke’s Cognitive Examination III (ACE-III) score’s ability to detect post-stroke cognitive impairment.

A total of 21/30 (70%) patients met criteria for PSCI during the acute stroke period according to the OCS, with a 95% confidence interval for the sample estimate ranging from 52.0 to 83.4%. An ROC curve for the total ACE-III score estimated that it could discriminate between normal cognition and PSCI as diagnosed by the OCS, with an AUC of 0.897 (95% CI 0.783–1.000) (*p* < 0.001) (Figure 1). Using the standard cut-off score of < 88/100, 20/30 (66.7%) patients were classified with PSCI, however 2/20 (10.0%) of these patients were found to be aphasic but cognitively intact by the OCS. Of the 10/30 (33.3%) patients classified with normal cognition, 3/10 (30.0%) were found to have PSCI according to the OCS. In comparison, 16/30 (53.3%) patients were classified with PSCI when using the proposed stroke-specific cut-off score of < 82/100, all of whom were also diagnosed with PSCI using the OCS. Of the 14/30 (46.7%) patients classified with normal cognition, 5/14 (35.7%) were found to have PSCI on the OCS.

The ACE-III failed to detect cognitive impairment in five patients who were diagnosed with PSCI on the OCS, four of whom (80.0%) demonstrated impairments in attention, two (40.0%) in memory, and one (20.0%) in language (Table 3). Among patients with impaired attention, 75% were due to specific impairments in complex attention and neglect, while 25% were from executive dysfunction.

**TABLE 3:** Patients with post-stroke cognitive impairment that were missed by the Addenbrooke’s Cognitive Examination III.

Patient	Attention	Language	Memory	Number	Praxis
1	Object neglect	–	–	–	–
2	–	–	Orientation	–	–
3	Cancellation object neglect	Sentence reading	–	–	–
4*	Cancellation	–	Verbal recall and recognition	–	–
5*	Executive function trails	–	–	–	–

*Patients missed with ACE-III < 82/100.

ROC curves were estimated for the remapped ACE-III domain sub-scores’ ability to detect domain-specific impairments as diagnosed by the OCS (Table 4). The AUC for the ACE-III attention sub-score’s ability to discriminate between normal and impaired attention was 0.770 (95% CI 0.603–0.937, *p* = 0.012), with an optimal cut-off of < 24.5/30. For discrimination between normal and abnormal language using the ACE-III language sub-score, the AUC was 0.914 (95% CI 0.793–1.000, *p* < 0.001) with an optimal cut-off of < 16.5/23. For the ACE-III memory sub-score’s ability to discriminate between normal and impaired memory, the AUC was 0.800 (95% CI 0.591–1.000, *p* = 0.005) with an optimal cut-off of < 26.5/39. The AUC for the ACE-III number processing sub-score’s ability to detect impaired number processing was 1.000 (*p* < 0.001), with an optimal cut-off of < 3.5/5. The ACE-III praxis sub-score showed poor discriminative performance, with an AUC of 0.547 (*p* = 0.734) and a suggested cut-off of < 1.5/3.

**TABLE 4:** Areas under the curve and optimal cut-offs for the remapped Addenbrooke’s Cognitive Examination III domain sub-scores.

Domain	AUC (95% CI)	*p*	Cut-off	*J*
Attention	0.770 (0.603–0.937)	0.012*	<24.5/30	0.411
Language	0.914 (0.793–1.00)	<0.001*	<16.5/23	0.806
Memory	0.800 (0.591–1.00)	0.005*	<26.5/39	0.700
Number	1.00 (1.00–1.00)	<0.001*	<3.5/5	0.950
Praxis	0.547 (0.277–0.816)	0.734	<1.5/3	0.242

AUC, area under curve; J, Youden index; 95% CI, 95% confidence interval. *p < 0.05.

Measures of diagnostic accuracy were calculated for the ACE-III’s standard and stroke-specific cut-off scores as well as for the remapped domain sub-scores that held discriminatory capacity for domain-specific impairments (Table 5).

**TABLE 5:** Accuracy of the Addenbrooke’s Cognitive Examination III’s total scores and domain-specific sub-scores in detecting post-stroke cognitive impairment.

Measure	88/100	82/100	Attention	Language	Memory	Number	Praxis
Sens, % (95% CI)	85.7 (63.7–97.0)	76.2 (52.8–91.8)	78.6 (49.2–95.3)	91.7 (61.5–99.8)	80.0 (44.4–97.5)	100.0 (69.2–100.0)	28.6 (3.7–71.0)
Spec, % (95% CI)	77.8 (40.0–97.2)	100.0 (66.4–100.0)	62.5 (35.4–84.8)	88.9 (65.3–98.6)	90.0 (68.3–98.8)	95.0 (75.1–99.9)	95.7 (78.1–99.9)
PLR (95% CI)	3.9 (1.1–13.3)	0	2.1 (1.1–4.2)	8.3 (2.2–30.8)	8.0 (2.1–30.9)	20.0 (3.0–135.1)	6.6 (0.7–62.1)
NLR (95% CI)	0.18 (0.06–0.55)	0.24 (0.11–0.51)	0.34 (0.12–1.0)	0.09 (0.01–0.62)	0.22 (0.06–0.77)	0	0.75 (0.46–1.20)
PPV% (95% CI)	90.0 (72.4–96.9)	100.0 (79.4–100.0)	83.0 (71.1–90.7)	95.1 (83.8–98.6)	94.9 (82.9–98.6)	97.9 (87.4–99.7)	93.9 (61.9–99.3)
NPV% (95% CI)	70.0 (43.6–87.6)	64.3 (45.6–79.5)	55.6 (30.0–78.5)	82.1 (41.0–96.8)	65.9 (35.6–87.1)	100.0 (82.4–100.0)	36.5 (26.3–48.0)

Sens, sensitivity; Spec, specificity; PLR, positive likelihood ratio; NLR, negative likelihood ratio; PPV, positive predictive value; NPV, negative predictive value; 95% CI, 95% confidence interval.

### 3.3 Domain-specificity of post-stroke cognitive impairment

Of the 21 patients diagnosed with PSCI using the OCS, 7/21 (33.3%) exhibited impairment in one domain, 5/21 (23.8%) in two domains, 4/21 (19.1%) in three domains, 1/21 (4.8%) in four domains, and 4/21 (19.1%) in all five domains. While severe PSCI (impairments in ≥ 3 domains) was associated with loss of independence (*p* = *0.030*), no patients met the diagnostic criteria for post-stroke dementia (Levine et al., 2015). Note that 4/30 (13.3%) patients had functional deficits which impacted their ability to be tested across all domains of the OCS, with significant upper limb weakness impacting three patients and cortical blindness impacting one patient.

The most commonly impaired cognitive domain was attention (46.7%), followed by language (40.0%), memory (33.3%), number processing (33.3%), and praxis (23.3%). Impairments in the praxis domain as well as tasks specifically assessing executive function and calculation were significantly associated with a loss of functional independence. Once adjusted for multiple comparisons, the difference between groups in the executive function task was not significant (Table 6).

**TABLE 6:** Distribution of domain-specific impairments by level of function.

OCS domain and tasks	All PSCI (*n* = 21) (%)	Independent (*n* = 11) (%)	Dependent (*n* = 10) (%)	*p*	Adjusted *p*
**Attention**	**14 (46.7)**	**7 (63.6)**	**7 (70.0)**	**1.000**	**1.000**
Cancellation	10 (33.3)	5 (45.5)	5 (50.0)	1.000	1.000
Object neglect	8 (26.7)	3 (27.3)	5 (50.0)	0.387	0.645
Spatial neglect	5 (16.7)	1 (9.1)	4 (40.0)	0.149	0.373
Executive function trails	7 (23.3)	1 (9.1)	6 (60.0)	0.024*	0.120
**Language**	**12 (40.0)**	**5 (45.5)**	**7 (70.0)**	**0.387**	**0.387**
Picture naming	6 (20.0)	1 (9.1)	5 (50.0)	0.063	0.252
Semantics	5 (16.7)	1 (9.1)	4 (40.0)	0.149	0.298
Sentence reading	12 (40.0)	5 (45.5)	7 (70.0)	0.387	0.387
**Memory**	**10 (33.3)**	**4 (36.4)**	**6 (60.0)**	**0.395**	**0.395**
Orientation	7 (23.3)	2 (18.2)	5 (50.0)	0.183	0.244
Verbal recall and recognition	8 (26.7)	2 (18.2)	6 (60.0)	0.080	0.180
Episodic recognition	3 (10.0)	0 (0)	3 (30.0)	0.090	0.180
**Number**	**10 (33.3)**	**3 (27.3)**	**7 (70.0)**	**0.086**	**0.086**
Number writing	10 (33.3)	3 (27.3)	7 (70.0)	0.086	0.086
Calculation	6 (20.0)	0 (0)	6 (60.0)	0.004*	0.012*
**Praxis**	**7 (23.3)**	**1 (9.1)**	**6 (60.0)**	0.024*	0.024*
Gesture imitation	7 (23.3)	1 (9.1)	6 (60.0)	0.024*	0.024*
**Severe PSCI**	**9 (42.9)**	**2 (18.2)**	**7 (70.0)**	0.030*	–

PSCI, post-stroke cognitive impairment; OCS, Oxford Cognitive Screen. *p < 0.05.

### 3.4 Sensitivity analysis

When the four patients who were unable to complete tests across all OCS domains were removed, PSCI prevalence was 65.4% (95% CI 46.3–80.7), and the ROC performance of the ACE-III was 0.873. For both ACE-III cut-offs, the differences in sensitivity before and after removal were 5.9 percentage points, with 95% CI for the difference crossing zero (< 88/100: -29.6 to 17.8%; < 82/100: -35.5 to 23.7%), and Cohen’s *h* < 0.16. Specificity did not change at either cut-off. Domain-level accuracy estimates followed a similar pattern, with small shifts and overlapping confidence intervals compared to the original analysis. Of note, the ACE-III praxis sub-score became non-discriminative (AUC = 0.364). The results of the sensitivity analysis are provided in Supplementary Tables 1, 2.

### 3.5 *Post-hoc* power analysis

Relative to a reference PSCI prevalence value of 40%, this sample provided approximately 91.8% power (two sided α = 0.05) to detect the higher PSCI prevalence observed, and 59.1% power to detect a prevalence higher than a threshold of 50%. In the sensitivity analysis cohort (removing the four impaired patients), a 75.1% power was retained to exceed the same 40% benchmark.

For the ACE-III total score, the observed AUC of 0.897 yielded a *post-hoc* power > 99.9% to detect discrimination better than chance. At the < 88/100 cut-off, the observed sensitivity among the impaired patients provided 67% power to exceed a 60% sensitivity threshold, and a 10% power to exceed a threshold of 80%. For specificity, power was 19% to distinguish the observed specificity from 60%, and 2% to distinguish it from 80%.

## 4 Discussion

This study provides the first comparison of the ACE-III and the OCS in an exclusively acute, first-ever stroke cohort free from prior cerebrovascular or cognitive disorders. Using the OCS as a stroke-specific reference tool, there was a high prevalence of PSCI. Although the ACE-III total score showed some discriminative value in identifying patients with PSCI, neither the standard nor stroke-specific cut-offs met recommended accuracy thresholds for cognitive screening after stroke. Only the remapped language and number-processing domain scores met minimum recommended criteria. Although limited by a small sample size, this exploratory study suggests that the ACE-III is insufficient as an early cognitive screening tool in acute stroke.

### 4.1 Validity of Addenbrooke’s Cognitive Examination in the acute stroke setting

While the ACE-III has been validated for the detection of non-vascular cognitive impairment and dementia, its validity for stroke-related impairments remains tenuous (Lees et al., 2017; Pendlebury et al., 2012). In our cohort, the standard cut-off score of 88/100 returned false positive results for two patients with aphasia. Concerns regarding the ACE-III’s suitability for assessing patients with aphasia have been reported in previous studies, and while this appears to be mitigated somewhat by the lower stroke-specific cut-off score of 82/100, there is a trade-off in terms of test specificity (Lees et al., 2017; Morris et al., 2012).

Our results demonstrated that the total ACE-III score held discriminatory capacity between normal cognition and PSCI in our cohort, as evidenced by the high AUC. However, when considering the minimum measures of accuracy for neurocognitive screening tools in stroke proposed by Stolwyk et al. (2014) (sensitivity ≥ 80%, specificity ≥ 60%, PPV ≥ 80%, and NPV ≥ 80%), both the standard and stroke-specific cut-off scores proved inadequate for the detection of PSCI. Additionally, individual domain performance was highly variable, illustrating the limited capacity of global cognitive screens to provide actionable, domain-level data. While the remapped ACE-III domain sub-scores for language and number processing both held discriminatory capacity and met the minimum standards for neurocognitive screening after stroke, their use might not be warranted given that the other components of the ACE-III are not clinically meaningful. Of note, the praxis domain demonstrated the weakest discriminative performance, likely reflecting the small number of patients with praxis impairment and therefore a stable distribution to allow for ROC estimation. As such, it appears that the ACE-III may not be adequate for the detection of PSCI in the acute stroke setting. These findings however must be validated in larger samples.

### 4.2 Post-stroke cognitive impairment after a first-ever stroke

The high incidence of PSCI identified in our cohort lies toward the upper range of previously reported acute incidence rates. Variability in reported incidence often results from differences in cognitive assessment tools. A recent study by Gallucci et al. (2024) similarly found that 69.3% of patients exhibited PSCI when assessed using a comprehensive neuropsychological battery, with attention being the most frequently impaired domain—a finding consistent with our results and other studies (Hurford et al., 2013; Leśniak et al., 2008). However only 44.1% of that cohort was classified as impaired when using the MoCA (Gallucci et al., 2024). This discrepancy underscores the limited sensitivity of global screening tools and highlights their inability to capture valuable domain-specific information critical for targeted rehabilitation planning. While detailed neuropsychological assessments are impractical in acute stroke settings, stroke-specific domain-focused tools like the OCS represent an optimal solution for routine screening. Supporting this, Milosevich et al. (2024) recently utilized the OCS in acute stroke patients, identifying cognitive impairment in 98.4% of participants, with 73.2% showing deficits across multiple domains. Although the inclusion of patients with recurrent strokes and pre-existing cognitive impairment likely contributed to their higher rates, their findings importantly demonstrated that acute cognitive function was the strongest predictor of cognitive performance at 6 months. In addition, domain-specific impairments in memory, language, and praxis were the best predictors of severity of cognitive impairment at follow-up. Collectively, these studies emphasize the clinical value and necessity of implementing early domain-specific cognitive screening in acute stroke care.

### 4.3 Identification and rehabilitation of domain-specific impairments

Early identification of domain-specific cognitive impairment is crucial for personalized rehabilitation and prognostication. In addition, contemporary rehabilitation approaches increasingly emphasize domain-specific interventions, with growing evidence supporting targeted therapies for distinct domain impairments (Basagni et al., 2024; Mulhern, 2023; O’Donoghue et al., 2022). In our cohort, deficits in praxis and number processing were notably associated with functional dependence, underscoring their potential prognostic significance. Both of these domains are also underrepresented in general cognitive screens. Severity of apraxia has been linked to early rehabilitation outcomes after stroke (Latarnik et al., 2022), and rehabilitation of limb apraxia through strategy and gesture training interventions improve motor function and activities of daily living (Alashram et al., 2022). In contrast, impairments in number processing and calculation are often neglected both in clinical practice and stroke research (Ardila and Rosselli, 2019). A recent qualitative study demonstrated impacts ranging from difficulty managing finances to medication and engaging in social activities and employment. While the OCS specifically looks for acalculia, further testing may be required to determine whether this impairment is primary or secondary to other cognitive impairments, as their management differs significantly. While less common, primary acalculia requires cognitive rehabilitation to rebuild patients’ understanding of numerical organization (Ardila and Rosselli, 2019). Most cases of secondary acalculia, on the other hand, recover in parallel to recovery of the underlying impairment and require no specific treatment (Basso et al., 2005).

The integration of neuromodulation techniques such as repetitive transcranial magnetic stimulation (rTMS) with domain-targeted cognitive rehabilitation holds particular promise by selectively modulating disrupted neural networks, potentially improving cognitive recovery beyond conventional rehabilitation approaches (Hofmeijer et al., 2023). However, more strides need to be made in determining the efficacy and utility of these techniques using randomized controlled trials in large cohorts (Hofmeijer et al., 2023; Zhao et al., 2024; Zhu et al., 2024).

### 4.4 Study limitations

This study has several important limitations. Recruitment was significantly impacted by the global COVID-19 pandemic, and, as a result, the study was inadequately powered for multivariate analysis to identify the contribution of clinical and stroke characteristics. Several domain-level analyses contained very few impaired participants, reducing precision and widening confidence intervals despite the *post-hoc* finding of adequate statistical power for some comparisons. The subgroup analyses should therefore be interpreted with caution. Second, although we applied a structured re-mapping of ACE-III items to OCS domains based on published frameworks and team consensus, this methodology has not been formally validated and may have influenced domain-level accuracy estimates. Third, the initial handling of untestable patients may introduce bias. This was examined through sensitivity analyses, which showed no material impact on the findings. Fourth, assessors were not blinded to the results of the alternative cognitive assessment, introducing the possibility of bias. Finally, patients were assessed only once during the acute hospital admission, and therefore the evolution of PSCI over time could not be evaluated. Future prospective studies should aim to recruit an adequately powered sample size to identify the impact of stroke characteristics and especially types of hyperacute treatment.

## 5 Conclusion

When compared with the OCS, the ACE-III demonstrated moderate ability in detecting overall PSCI. However, it may not be accurate for clinical use in the acute stroke setting, especially in assessing domain-specific dysfunction. The OCS is beneficial for the early identification of patients with specific impairments that may be amenable to early intervention, including patients at increased risk of developing dementia. This could ultimately lead to improved post-stroke functional recovery.

## Data Availability

The raw data supporting the conclusions of this article will be made available by the authors, without undue reservation.
