# Supplementary material for: Domain-specific cognitive screening in acute first-ever stroke: a comparative study of the Oxford Cognitive Screen (OCS) and ACE-III
Source: Front Hum Neurosci. 2026 Jan 12;19:1678230. doi: 10.3389/fnhum.2025.1678230 (PMC12833042; doi:10.3389/fnhum.2025.1678230)
Supplement: Supplementary file 1 [file Table_1.docx]

Supplementary Table 1: Areas under the curve and optimal cut-offs for the remapped Addenbrooke’s Cognitive Examination III domain sub-scores for the cohort of 26 patients (following removal of four patients with indeterminate results)

|  | **AUC (95% CI)** | ***p*** | **Cut-off** | **J** |
| --- | --- | --- | --- | --- |
| **Attention** | 0.752 (0.564 – 0.939) | 0.008 | < 25.5 | 0.418 |
| **Language** | 0.872 (0.697 – 1.000) | < 0.001 | < 16.5 | 0.764 |
| **Memory** | 0.675 (0.368 – 0.982) | 0.264 | < 26.5 | 0.567 |
| **Number** | 1.000 (1.000 – 1.000) | < 0.001 | < 2.5 | 1.000 |
| **Praxis** | 0.364 (0.109 – 0.618) | 0.294 | - | - |

^(AUC = area under curve, J = Youden index, 95% CI = 95% confidence interval)^

Supplementary Table 2: Accuracy of the Addenbrooke’s Cognitive Examination III’s total scores and domain-specific sub-scores in detecting post-stroke cognitive impairment for the cohort of 26 patients (following removal of four patients with indeterminate results)

|  | **88/100** | **82/100** | **Attention** | **Language** | **Memory** | **Number** |
| --- | --- | --- | --- | --- | --- | --- |
| **Sens (%)** | 82.4 (56.6 – 96.2) | 70.6 (44.0 – 89.7) | 81.8 (48.2 – 97.7) | 87.5 (47.4 – 99.7) | 66.7 (22.3 – 95.7) | 100.0 (54.1 – 100.0) |
| **Spec (%)** | 77.8 (40.0 – 97.2) | 100.0 (66.4 – 100.0) | 60.0 (32.3 – 83.7) | 88.9 (65.3 – 98.6) | 90.0 (68.3 – 98.8) | 100.0 (83.2 – 100.0) |
| **PLR** | 3.71 (1.07 – 12.83) | - | 2.05 (1.04 – 4.04) | 7.87 (2.08 – 29.85) | 6.67 (1.59 – 27.90) | - |
| **NLR** | 0.23 (0.08 – 0.67) | 0.29 (0.14 – 0.61) | 0.30 (0.08 – 1.13) | 0.14 (0.02 – 0.89) | 0.37 (0.12 – 1.16) | 0.0 |
| **PPV (%)** | 87.5 (66.9 – 96.0) | 100.0 (73.5 – 100.0) | 79.5 (66.2 – 88.4) | 93.7 (79.7 – 98.3) | 92.7 (75.1 – 98.1) | 100.0 (54.1 – 100.0) |
| **NPV (%)** | 70.0 (44.1 – 87.4) | 60.7 – 93.5) | 63.6 (31.8 – 86.7) | 79.0 (37.4 – 96.0) | 58.8 (31.3 – 81.7) | 100.0 (83.2 – 100.0) |

^(Sens = sensitivity, Spec = specificity, PLR = positive likelihood ratio, NLR = negative likelihood ratio, PPV = positive predictive value, NPV = negative predictive value, 95% CI = 95% confidence interval)^
